# Neofunctionalization of *S*-adenosylmethionine decarboxylase into pyruvoyl-dependent L-ornithine and L-arginine decarboxylases is widespread in bacteria and archaea

**DOI:** 10.1016/j.jbc.2023.105005

**Published:** 2023-07-01

**Authors:** Bin Li, Jue Liang, Margaret A. Phillips, Anthony J. Michael

**Affiliations:** Department of Biochemistry, UT Southwestern Medical Center, Dallas, Texas, USA

**Keywords:** polyamine, *S*-adenosylmethionine, ornithine, arginine, decarboxylase, neofunctionalization, putrescine, agmatine, pyruvoyl

## Abstract

*S*-adenosylmethionine decarboxylase (AdoMetDC/SpeD) is a key polyamine biosynthetic enzyme required for conversion of putrescine to spermidine. Autocatalytic self-processing of the AdoMetDC/SpeD proenzyme generates a pyruvoyl cofactor from an internal serine. Recently, we discovered that diverse bacteriophages encode AdoMetDC/SpeD homologs that lack AdoMetDC activity and instead decarboxylate L-ornithine or L-arginine. We reasoned that neofunctionalized AdoMetDC/SpeD homologs were unlikely to have emerged in bacteriophages and were probably acquired from ancestral bacterial hosts. To test this hypothesis, we sought to identify candidate AdoMetDC/SpeD homologs encoding L-ornithine and L-arginine decarboxylases in bacteria and archaea. We searched for the anomalous presence of AdoMetDC/SpeD homologs in the absence of its obligatory partner enzyme spermidine synthase, or the presence of two AdoMetDC/SpeD homologs encoded in the same genome. Biochemical characterization of candidate neofunctionalized genes confirmed lack of AdoMetDC activity, and functional presence of L-ornithine or L-arginine decarboxylase activity in proteins from phyla Actinomycetota, Armatimonadota, Planctomycetota, Melainabacteria, Perigrinibacteria, Atribacteria, Chloroflexota, Sumerlaeota, Omnitrophota, Lentisphaerota, and Euryarchaeota, the bacterial candidate phyla radiation and DPANN archaea, and the δ-Proteobacteria class. Phylogenetic analysis indicated that L-arginine decarboxylases emerged at least three times from AdoMetDC/SpeD, whereas L-ornithine decarboxylases arose only once, potentially from the AdoMetDC/SpeD-derived L-arginine decarboxylases, revealing unsuspected polyamine metabolic plasticity. Horizontal transfer of the neofunctionalized genes appears to be the more prevalent mode of dissemination. We identified fusion proteins of *bona fide* AdoMetDC/SpeD with homologous L-ornithine decarboxylases that possess two, unprecedented internal protein-derived pyruvoyl cofactors. These fusion proteins suggest a plausible model for the evolution of the eukaryotic AdoMetDC.

One of the ways in which new enzyme activities can emerge is by evolution from preexisting enzymes that harbor a latent promiscuity toward noncanonical substrates (1). A promiscuous activity can become selected for after gene duplication if it provides an adaptive advantage. After gene duplication, the original enzyme activity is retained in one copy and a new activity emerges in the other if there is selective pressure for the promiscuous activity, a process termed neofunctionalization (1, 2, 3, 4). New enzymes emerging by neofunctionalization must then be integrated into existing metabolic networks or provide a novel physiological adaptation. In bacteria and archaea, an additional contribution to acquisition of new enzyme activities comes from horizontal gene transfer (HGT) (3), which may be up to 50-fold more frequent than gene duplication (5). The emergence of new enzyme activities from preexisting ones has played a major role in the expansion of the original enzyme complement of last universal common ancestor, that is, just 500 genes (6) or 355 protein families (7).

The small, linear, and primordial polyamine spermidine (Fig. 1) is the most common polyamine found in bacteria, archaea, and eukaryotes (8). Its role in bacterial growth and proliferation under laboratory growth conditions ranges from being absolutely essential to being entirely dispensable (9). Spermidine is synthesized from the simple diamine putrescine (1,4-diaminobutane) by transfer of an aminopropyl group from decarboxylated *S*-adenosylmethionine (dcAdoMet) to putrescine by spermidine synthase (SpdSyn/SpeE) (Fig. 1). The dcAdoMet is formed from AdoMet by AdoMet decarboxylase (AdoMetDC/SpeD), a protein belonging to a small group of enzymes that have bypassed the requirement for a separate cofactor by generating a pyruvoyl cofactor from a serine residue in their own polypeptide chain (10). The proenzyme form of AdoMetDC/SpeD undergoes an autocatalytic processing reaction to produce new α- and β-subunits, with the internal serine-derived pyruvoyl cofactor at the N-terminus of the α-subunit (11).

**Figure 1:**
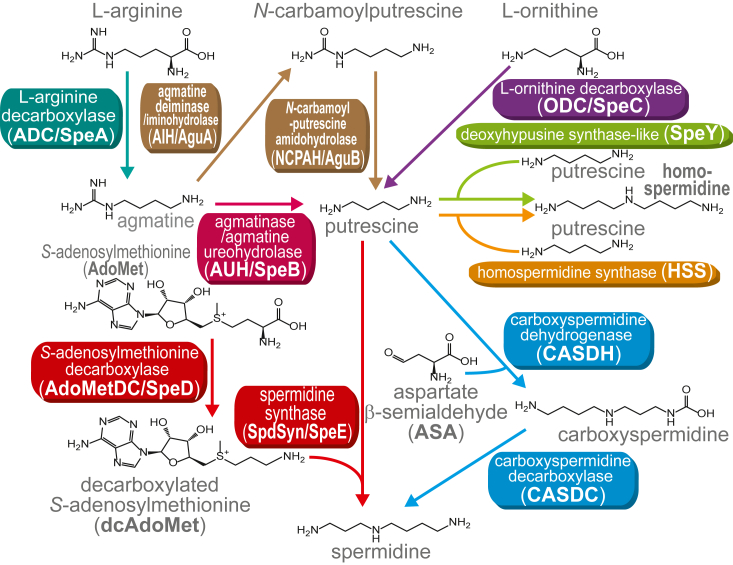
**Polyamine biosynthetic pathways.** Enzymes of the same biosynthetic module are shown in the same color.

Two homologous forms of AdoMetDC/SpeD exist in bacteria: class 1b and 1a, with the 1a form being derived from the smaller 1b form through amino acid insertions and extensions (12). Eukaryotic AdoMetDC (class 2), while exhibiting essentially little to no detectable sequence homology to the prokaryotic class 1 forms, was found from structural analysis to consist of two fused class 1b proteins (12). The putrescine required for spermidine biosynthesis can be produced indirectly from L-arginine or directly from L-ornithine by L-arginine and L-ornithine decarboxylases, respectively (L-ADC/SpeA and L-ODC/SpeC) (13). Putrescine is also the precursor for biosynthesis of the spermidine structural analog *sym*-homospermidine (14, 15) (Fig. 1).

Polyamine metabolism has diverse examples of the emergence of new polyamine biosynthetic enzymes from preexisting polyamine-related enzymes, that is, neofunctionalization within the same metabolic pathway (16). For example, spermine synthase has evolved from spermidine synthase (SpdSyn) in flowering plants (17) and in Saccharomycotina yeasts (18). An alanine racemase fold pyridoxal-5'-phosphate (PLP)-dependent ODC homolog of the chlorovirus *Paramecium bursaria* chlorella virus 1 was found to be an ADC rather than an ODC (19). Similar ODC homologs in the α-proteobacteria *Candidatus Pelagibacter ubique* and *Ca*. *Fonsibacter ubiquis* also exhibit ADC rather than ODC activity (20). An archaeal class 1b AdoMetDC/SpeD homolog in the Crenarchaeote *Sulfolobus solfataricus* was found to have lost AdoMetDC activity but instead exhibits ADC activity (21).

We recently identified class 1b AdoMetDC/SpeD homologs encoded by different bacteriophages that have lost AdoMetDC activity but exhibit either ADC or ODC activity (20). Either these ADC and ODC activities evolved from the SpeD homologs while encoded by the bacteriophage, or the previously neofunctionalized genes were acquired by the bacteriophages from ancestral bacterial hosts. Neofunctionalized AdoMetDC/SpeD proteins have not yet been reported in bacteria. We sought to systematically identify *speD* genes encoded in bacterial and archaeal genomes that might encode ADC or ODC rather than AdoMetDC activities. To do this, we searched for *speD* homologs that were either present in genomes in the absence of SpdSyn/*speE*, and so would be unlikely to be involved in the biosynthesis of spermidine from putrescine, or were present in addition to another *speD* homolog with *speE*, suggesting that one *speD* homolog may encode a function different to AdoMetDC. A phylogenetically broad selection of candidate genes was biochemically characterized, identifying multiple cases of pyruvoyl-dependent ADCs and ODCs in bacteria and archaea. Furthermore, we were able to construct a plausible scenario for the evolution of the eukaryotic class 2 AdoMetDC from a fusion of a *bona fide* class 1b bacterial *speD* gene and a degraded *speD* homologous pyruvoyl-dependent ODC.

## Results

**Figure 2:**
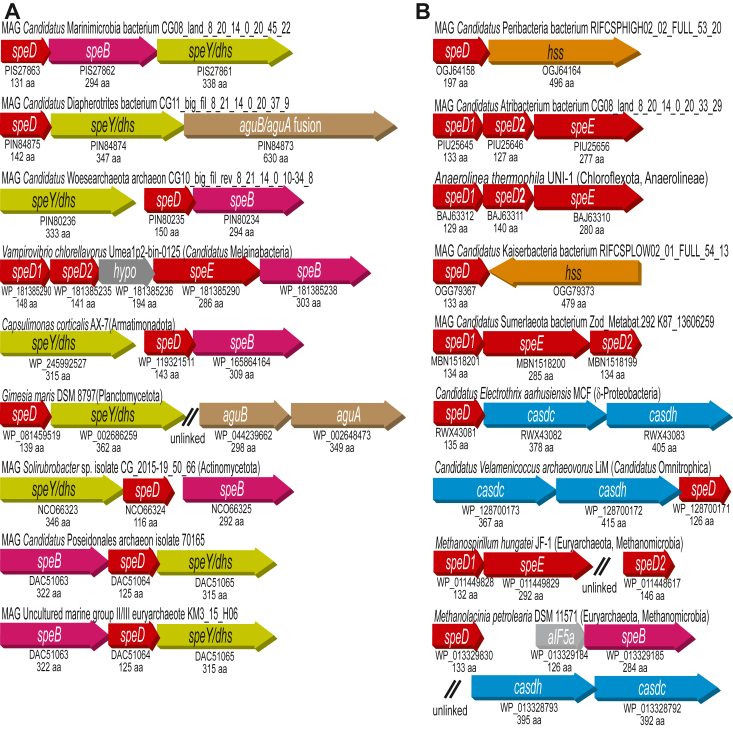
**Bacterial and archaeal gene clusters encoding polyamine biosynthetic enzymes.***A*, gene clusters containing agmatine ureohydrolase or agmatine deiminase and *N*-carbamoylputrescine amidohydrolase for conversion of agmatine to putrescine. *B*, gene clusters from genomes lacking agmatine ureohydrolase or agmatine deiminase and *N*-carbamoylputrescine amidohydrolase.

Bacteria and archaea each encode alternative biosynthetic pathways for both spermidine and homospermidine production (13) (Fig. 1). Spermidine is synthesized by either AdoMetDC/SpdSyn (SpeD/SpeE) or by carboxyspermidine dehydrogenase/decarboxylase (CASDH/CASDC); homospermidine is synthesized by either homospermidine synthase (HSS) or a deoxyhypusine synthase homolog (SpeY). We identified the anomalous presence of SpeD homologs encoded in diverse bacterial and archaeal genomes in the absence of a SpeE homolog. Some genomes encode two SpeD homologs but only one SpeE. Selected examples of polyamine biosynthetic gene clusters encoding anomalous SpeD homologs are shown in Figure 2. Anomalous SpeD homologs are encoded in gene clusters together with either SpeD/SpeE, CASDH/CASDC, HSS, or SpeY. We also noticed that all genomes encoding an anomalous AdoMetDC homolog also lacked canonical ADC (SpeA) or ODC (SpeC) for putrescine biosynthesis. Some genomes encoding an anomalous SpeD also encode agmatine ureohydrolase (agmatinase, AUH/SpeB) for direct conversion of agmatine to putrescine. Others encode agmatine deiminase (AIH/AguA) and *N*-carbamoylputrescine amidohydrolase (NCPAH/AguB) for conversion of agmatine to putrescine *via N*-carbamoylputrescine (Figs. 1 and 2). The presence of either SpeB or AguA/AguB encoded in some genomes that contain anomalous *speD* suggested that the anomalous *speD* gene may encode ADC activity. The corollary of this suggestion is that in genomes not encoding SpeB or AguA/AguB, the anomalous *speD* gene may encode ODC.

**Figure 3:**
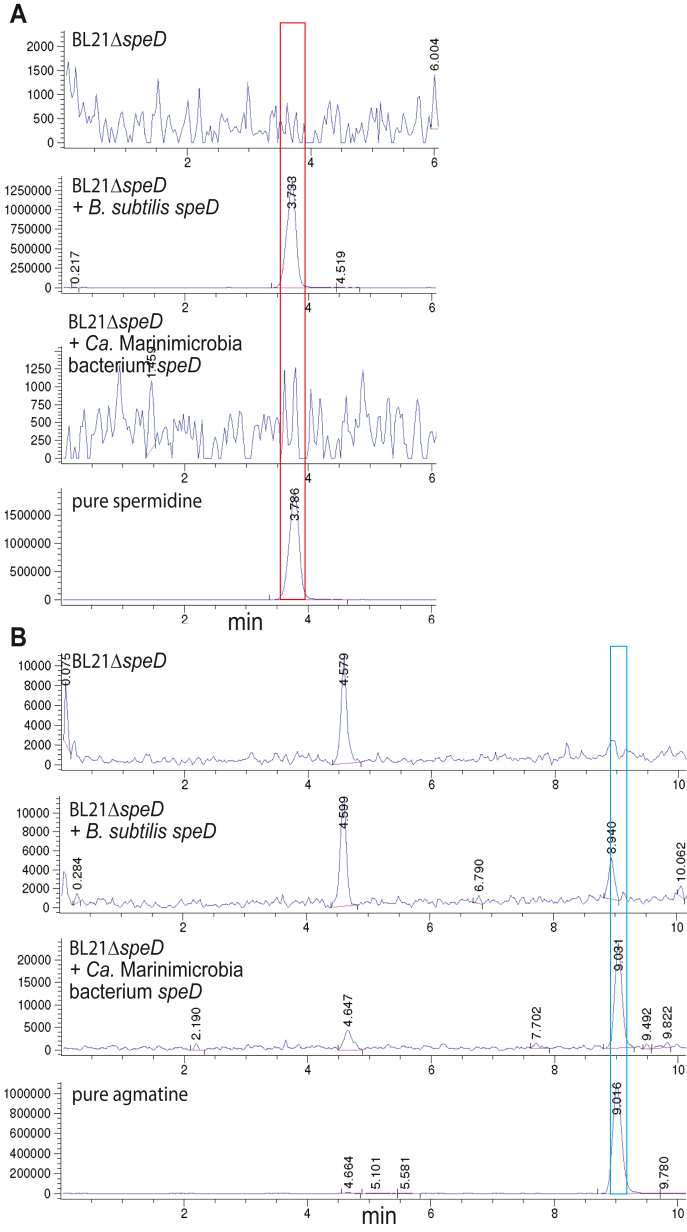
**LC-MS analysis of benzoylated extracts of *Escherichia coli* BL21Δ*speD* expressing *speD* homologues.** Benzoylated extracts of *E. coli* BL21Δ*speD*, an *E. coli* strain lacking *S*-adenosylmethionine decarboxylase (*speD*), expressing either an empty pETDuet-1 plasmid, the *Bacillus subtilis speD* gene (encoding AQZ91791), or the *Ca.* Marinibacteria bacterium *speD* (encoding PIS27863). *A*, extracted ion chromatograms (EICs) for tribenzoylated spermidine (EIC, 457.9:458.9). *B*, extracted ion chromatograms (EICs) for tribenzoylated agmatine (EIC, 442.9:443.9).

To test the hypothesis that some *speD* homologues may encode ADC, we chose to investigate the *speD* gene found in a metagenome-assembled genome (MAG) from the candidate bacterial phylum Marinimicrobia. The *Candidatus* Marinimicrobia bacterium genome was obtained from terrestrial deep subsurface water (22) and contains a gene cluster encoding SpeD, SpeB, and SpeY for homospermidine biosynthesis (Fig. 2*A*). We employed a spermidine-deficient *speD* gene deletion strain of *Escherichia coli* (BL21*speD*) to express the *Ca*. Marinimicrobia *speD*, using the *Bacillus subtilis speD* as a positive control (Fig. 3*A*). Whereas the *B. subtilis speD* gene restored spermidine biosynthesis, detected as the tribenzoylated product by LC-MS, the *Ca*. Marinimicrobia *speD* gene did not. Failure to restore spermidine biosynthesis could have been due to the lack of expression of the *Ca*. Marinimicrobia bacterium *speD* gene. Therefore, we also looked at agmatine production (*i.e.*, the product of arginine decarboxylation) and found a large accumulation of agmatine only in the BL21*speD* strain expressing the *Ca*. Marinimicrobia bacterium *speD* gene (Fig. 3*B*).

**Figure 4:**
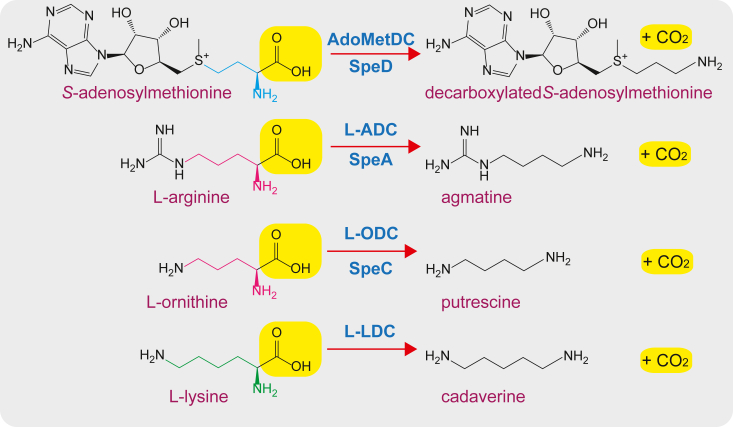
**Polyamine-related decarboxylase reactions.** Enzymes shown are AdoMetDC/SpeD, *S*-adenosylmethionine decarboxylase; L-ADC/SpeA, L-arginine decarboxylase; L-ODC/SpeC, L-ornithine decarboxylase; L-LDC, L-lysine decarboxylase.

The *Ca*. Marinimicrobia *speD* gene was expressed in *E. coli* BL21, the recombinant protein purified, and its enzymatic activity subjected to kinetic analysis using L-arginine, L-ornithine, or L-lysine as substrates (substrates and products shown in Figure 4). Using a coupled assay for CO_2_ release detection, no activity was detected with L-ornithine or L-lysine but with L-arginine the enzyme exhibited a *k*_cat_/*K*_m_ of 770 ± 37 M^−1^ s^−1^ (Table 1). This is the first bacterial SpeD homolog, that is, *S*-adenosylmethionine decarboxylase homolog, functionally proven to exhibit ADC activity. To cross-validate the ADC activity of the *Ca*. Marinimicrobia SpeD protein, the purified protein was incubated with 10 mM L-arginine and the reaction products subjected to LC-MS analysis (Fig. S1). Some agmatine carried over from *E. coli* in the purified protein preparation; however, the functional enzyme produced more than 3-fold more agmatine than the boiled enzyme and 600-fold more agmatine than the enzyme without L-arginine. This suggests that the boiled enzyme still retained some activity, as the L-arginine stock did not contain any detectable agmatine.

**Table 1:** Kinetic constants determined for AdoMetDC homologues

Species	Substrate	*K*_m_ (mM)	*k*_cat_ (s^−1^)	*k*_cat_/*K*_m_ (M^−1^s^−1^)
*Ca*. Marinimicrobia bacterium	L-ornithine	ND.	ND	ND
*Ca*. Marinimicrobia bacterium	L-arginine	0.76 ± 0.050	0.58 ± 0.010	770 ± 37
*Ca*. Marinimicrobia bacterium	L-lysine	ND	ND	ND
*Ca*. Peribacteria bacterium	L-ornithine	0.27 ± 0.020	0.17 ± 0.0050	620 ± 26
*Ca*. Peribacteria bacterium	L-arginine	ND	ND	ND
*Ca*. Peribacteria bacterium	L-lysine	ND	ND	ND
*Ca*. Atribacteria bacterium	L-ornithine	0.33 ± 0.030	0.27 ± 0.0070	820 ± 61
*Ca*. Atribacteria bacterium	L-arginine	ND	ND	ND
*Ca*. Atribacteria bacterium	L-lysine	ND	ND	ND
*Anaerolinea thermophila* UNI-1	L-ornithine	0.34 ± 0.030	0.12 ± 0.0020	580 ± 44
*Anaerolinea thermophila* UNI-1	L-arginine	ND	ND	ND
*Anaerolinea thermophila* UNI-1	L-lysine	ND	ND	ND

*Ca*. Marinimicrobia bacterium CG08_8_20_14_0_20_45_22 [GenBank protein accession no. PIS27863] 131 a.a., *Ca*. Peribacteria bacterium RIFCSPHIGH02_02_FULL_53_20 [OGJ64158] 197 a.a., *Ca*. Atribacteria bacterium CG08_land_8_20_14_0_20_33_29 [PIU25646] 127 a.a., *Anaerolinea thermophila* UNI-1 (Chloroflexota, Anaerolineae) [BAJ63311] 140 a.a. ND, no detectable activity. All assays performed in triplicate at 26 °C (mean ± S.D.).

Anomalous *speD* genes found in genomes that do not encode a biosynthetic route from agmatine to putrescine potentially encode ODC activity, converting L-ornithine directly to putrescine. To test this hypothesis, we purified proteins encoded by three different MAGs. One SpeD homolog is encoded by a genome from the candidate phylum Peregrinibacteria, *Ca*. Peribacteria bacterium (23) isolated from an aquifer groundwater metagenome. This genome contains a gene cluster encoding SpeD and HSS (Fig. 2*B*). Another SpeD homolog is encoded by the candidate phylum Atribacteria, *Ca*. Atribacteria bacterium obtained from terrestrial deep subsurface water (22), and is found in a gene cluster with a second *speD* homolog and *speE*. The third anomalous SpeD homolog is encoded by a Chloroflexota bacterium *Anaerolinea thermophila* UNI-1, a thermophilic anaerobic multicellular filamentous species isolated from wastewater sludge (24). This genome contains a gene cluster encoding a second SpeD homolog and SpeE. The *Ca*. Atribacteria bacterium and *A. thermophila* UNI-1 SpeD homologs with greatest amino acid identity to the Peribacteria bacterium singleton SpeD were chosen for *in vitro* analysis (SpeD2 in each case, Fig. 2*B*). Purified proteins encoded by the anomalous *speD* genes exhibited no detectable activity with L-arginine or L-lysine but were active on L-ornithine with *k*_cat_/*K*_m_ values ranging from 580 to 820 M^−1^ s^−1^ (Table 1). These are the first bacterial pyruvoyl-dependent ODCs to be functionally identified. The gene nomenclature for prokaryotic ADC- and ODC-encoding genes is *speA* and *speC*, respectively. We propose to name the *speD* homologues encoding ADC or ODC activity as *speDA* and *speDC*, respectively. Based on our trial analysis of the *Ca*. Marinibacteria, *Ca*. Peribacteria, *Ca*. Atribacteria and *A. thermophila* UNI-1 anomalous SpeD homologs, we proceeded to analyze *speD* homologs from diverse bacteria and archaea shown in Figure 2, and in addition, two homologs encoded by eukaryotes.

### SpeD homologs exhibiting ADC activity (SpeDA)

The *speDA* gene candidate from marine MAG uncultured marine group II/III euryarchaeota KM3_15_H06 is found clustered with *speB* and *speY* (Fig. 2*A*). An identical gene arrangement is found in marine MAG *Ca*. Poseidonales archaeon isolate 70165 (25). A very similar potential SpeDA protein is encoded by an intronized gene in the eukaryotic oleaginous model alga *Nannochloropsis gaditana* (26) from the Stramenopiles phylum. Similarly, a relatively close homolog is found encoded by an intronized gene in the eukaryotic stramenopile yellow green alga *Tribonema minus*, which was isolated from a wastewater treatment plant (27). A potential *speDA* gene is found in the marine planctomycetota bacterium *Gimesia maris* (formerly *Planctomyces maris* (28)) that is immediately upstream of *speY*, with *aguA* and *aguB* found clustered together elsewhere in the genome. Candidate *speDA* genes are also found in archaeal MAGs from terrestrial deep subsurface water: *Ca*. Diapherotrites archaeon and *Ca*. Woesearchaeota archaeon from the archaeal DPANN superphylum (22). The *Ca*. Diapherotrites archaeon potential *speDA* gene is found clustered with *speY* and a fused *aguA-aguB* gene, whereas the *Ca*. Woesearchaeota archaeon *speDA* candidate is clustered with *speY* and *speB*. Another *speDA* candidate is found in the actinobacterota *Solirubrobacter* sp., also isolated from deep subsurface water (29), and is found clustered with *speY* and *speB*. A *speDA* candidate in *Capsulimonas corticalis* (Armatimonadota phylum) isolated from the trunk surface of a Japanese beech tree (30) is found clustered with *speY* and *speB*. Two *speD* homologous genes, clustered with *speE* and *speB* are found in the predatory bacterium *Vampirovibrio chlorellavorus* (*Ca*. Melainabacteria phylum), which feeds on species of the chlorophyte alga *Chlorella* (31).

A selection of the candidate *speDA* homologs was expressed in the spermidine-deficient *E. coli* BL21*speD* strain to assess whether they could restore spermidine biosynthesis (Figs. S2 and S3). Although the *B. subtilis speD* gene restored spermidine biosynthesis, the homologs from *Ca*. Poseidonales archaeon, uncultured euryarchaeota marine group II/III, *Solirubrobacter* sp., and *C. corticalis* did not (Fig. S2). Of the two *V. chlorellavorus speD* homologs, the gene encoding protein WP_181385290 (SpeD1) restored spermidine biosynthesis but WP_181385235 (SpeD2) did not (Fig. S3), suggesting that *speD2* is the potential *speDA* gene. All of the potential *speDA* genes shown in Figure 2*A*, plus the eukaryotic homologs from *N. gaditana* and *T. minus* were expressed in *E. coli* BL21 and the encoded proteins purified. Each protein was assayed with L-arginine, L-ornithine, and L-lysine; none of the proteins were detectably active with L-ornithine or L-lysine but all were active with L-arginine (Table 2). A large range in *k*_cat_/*K*_m_ values was observed with the different proteins (approximately 100-fold), with the marine species exhibiting particularly low activities, and DPANN archaea among the highest.

**Table 2:** Kinetic constants determined for AdoMetDC (SpeD) homologues assayed with L-arginine

Species	protein	*K*_m_ (mM)	*k*_cat_ (s^−1^)	*k*_cat_/*K*_m_ (M^−1^s^−1^)
*Ca*. Diapherotrites archaeon	PIN84875	0.095 ± 0.019	0.21 ± 0.028	2200 ± 180
*Ca*. Woesearchaeota archaeon	PIN80235	0.35 ± 0.024	0.20 ± 0.0060	600 ± 35
*Vampirovibrio chlorellavorus*	WP_181385235	0.26 ± 0.021	0.20 ± 0.0040	780 ± 47
*Capsulimonas corticalis*	WP_119321511	0.37 ± 0.050	0.080 ± 0.0030	220 ± 36
*Ca*. Poseidonales archaeon	DAC51064	2.8 ± 0.50	0.48 ± 0.042	180 ± 19
Unc. marine group II/III euryarchaeota	AIF02979	2.1 ± 0.91	0.12 ± 0.017	64 ± 23
*Gimesia maris*	WP_081459519	3.4 ± 0.77	0.37 ± 0.020	110 ± 18
*Solirubrobacter* sp.	NCO66324	1.1 ± 0.44	0.019 ± 0.0010	20 ± 7.3
*Tribonema minus*	KAG5182568	0.72 ± 0.11	0.080 ± 0.0040	110 ± 10
*Nannochloropsis gaditana*	XP_005856029	1.3 ± 0.10	0.049 ± 0.0030	39 ± 0.30

No activity was detected with L-ornithine or L-lysine as substrates. All assays performed in triplicate at 26 °C (mean ± S.D.).

### SpeD homologs exhibiting ODC activity (SpeDC)

Of the anomalous *speD* homologs encoding potential SpeDC proteins shown in Figure 2*B*, three are encoded by archaeal species from the Methanomicrobia class of the Euryarchaeota phylum. *Methanolacinia petrolearia* (32) was isolated from an offshore oil field, and unclustered genes encode SpeD, SpeB, CASDH, and CASDC. *Methanogenium cariaci* is a marine species isolated from the sediment in a deep-sea trench (33), and *Methanospirillum hungatei* was isolated from an anaerobic sewage treatment digestor (34) and encodes a second SpeD homolog and SpeE. A potential *speDC* gene is found in *Ca*. *Velamenicoccus archaeovorus* LiM, a member of the bacterial candidate phylum Omnitrophota, and this bacterium appears to metabolically predate on *Methanosaeta* archaea (35). Another SpeDC candidate is encoded by a MAG from a subsurface metagenome found in an aquifer system and belongs to the candidate phyla radiation: *Ca*. Kaiserbacteria bacterium (36). It contains a *hss* gene clustered in the opposite direction to the potential *speDC* gene. Two SpeD homologs are encoded by a freshwater sediment MAG: *Ca*. Sumerlaeota bacterium of the Sumerlaeota candidate phylum (37), clustered with *speE*. Finally, one homolog is encoded by *Ca*. *Electrothrix aarhusiensis*, a δ-proteobacterium that forms centimeter-long multicellular filaments (38). The potential *speDC* gene is immediately upstream of *casdc* and *casdh*.

Genes encoding potential SpeDC proteins were expressed in the spermidine-deficient *E. coli* BL21*speD* strain. None of the singleton candidate *speDC* genes were able to restore spermidine biosynthesis (*Ca*. Kaiserbacteria bacterium, *Ca*. *E. aarhusiensis*, *Ca. V. archaeovorus* LiM Fig. S2; *M. petrolearia* and *M. cariaci*, Fig. S4). However, one SpeD homolog encoded by each pair of genes (*speD1*, Fig. 2*B*) from *Ca*. Sumerlaeota bacterium (MBN1518201, Fig. S3) and *M. hungatei* (WP_011449828, Fig. S4) restored spermidine biosynthesis, confirming *bona fide* AdoMetDC activity. Genes that failed to restore spermidine biosynthesis to *E. coli* BL21*speD* were expressed in *E. coli* BL21 and the encoded proteins (including SpeD2, MBN1518199, *Ca*. Sumerlaeota bacterium; SpeD2, WP011448617, *M. hungatei*) purified. None of the proteins exhibited detectable decarboxylase activity with L-arginine or L-lysine; however, all proteins were active with L-ornithine (Table 3). The *k*_cat_/*K*_m_ values for each protein with L-arginine ranged by 10-fold, from 113 to 1100 M^−1^ s^−1^, with *k*_cat_ values for some proteins being particularly low, although there appears to be compensation by lower *K*_m_ values. The Methanomicrobia SpeDC homologs are the first examples of pyruvoyl-dependent ODCs identified in archaea.

**Table 3:** Kinetic constants determined for AdoMetDC (SpeD) homologues assayed with L-ornithine

Species	Protein	*K*_m_ (mM)	*k*_cat_ (s^−1^)	*k*_cat_/*K*_m_ (M^−1^s^−1^)
*Ca*. Kaiserbacteria bacterium	OGG79367	0.056 ± 0.002	0.061 ± 0.003	1100 ± 58
*Methanogenium cariaci* JCM 10550	WP_062398326	0.044 ± 0.003	0.041 ± 0.002	920 ± 31
*Ca.* Sumerlaeota bacterium	MBN1518199	0.021 ± 0.0013	0.010 ± 0.0010	470 ± 78
*Ca. Electrothrix aarhusiensis*	RWX43081	0.089 ± 0.013	0.030 ± 0.0030	340 ± 20
*Methanolacinia petrolearia DSM 11571*	WP_048130809	0.24 ± 0.002	0.057 ± 0.002	240 ± 11
*Methanospirillum hungatei* JF-1	WP_011448617	0.23 ± 0.018	0.050 ± 0.0002	220 ± 18
*Ca. Velamenicoccus archaeovorus* LiM	WP_128700171	0.073 ± 0.0032	0.008 ± 0.0004	113 ± 20

No activity was detected with L-arginine or L-lysine as substrates. All assays performed in triplicate at 26 °C (mean ± S.D.).

### Fusion proteins of two SpeD homologs

**Figure 5:**
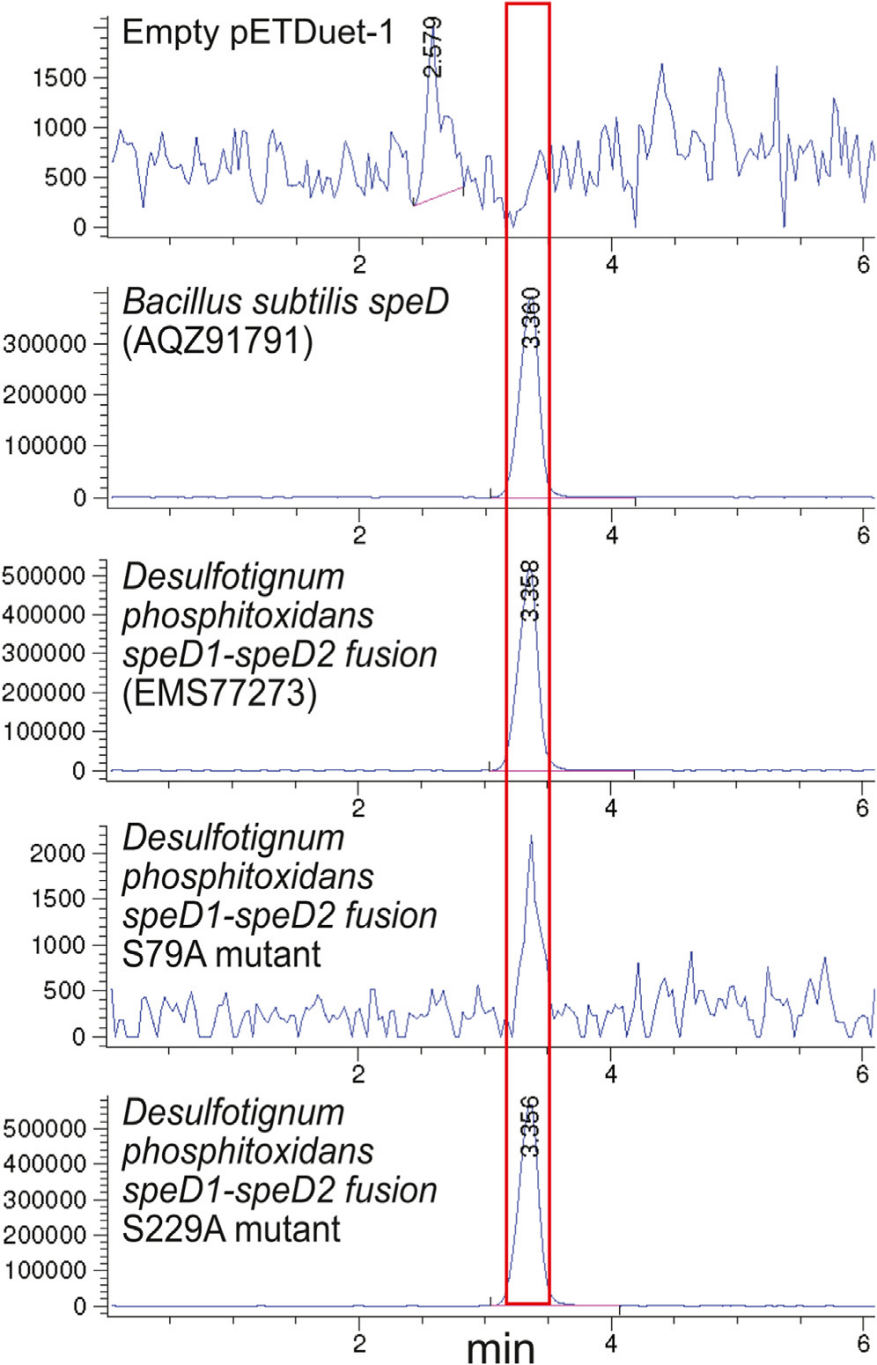
**LC-MS analysis of *Escherichia coli* BL21Δ*speD* benzoylated cell extracts.** Extracted ion chromatograms (457.94:458.94) for detection of tribenzoylated spermidine. Spermidine-deficient *E. coli* BL21Δ*speD* expressed the indicated genes from pETDuet-1 after growth in polyamine-free M9 chemically defined medium. The peak for the mass of tribenzoylated spermidine is outlined in *red*.

We noticed that some bacterial genomes encode a single ORF consisting of a fusion of two *speD* homologous genes located immediately upstream of a *speE* gene but with no other polyamine biosynthetic genes present in the genome. This suggested that the fused *speD1speD2* ORF might consist of a *bona fide speD* fused to a *speDC* gene, which together with the SpeE-encoding ORF would allow the biosynthesis of putrescine and spermidine from L-ornithine. The *speD1speD2* fusion ORFs from *Ca*. Omnitrophica bacterium OLB16 [KXK35843; 318 aa] and the δ-proteobacterium (now transferred to Thermodesulfobacteriota (39)) *Desulfotignum phosphitoxidans* DSM 13687 [WP_006968726; 278 aa] were selected for expression in spermidine-deficient *E. coli* BL21*speD*. There are also numerous similar fusion proteins encoded by Lentisphaerota bacteria. However, many of the Lentisphaerota fusion proteins lack key active site residues in the C-terminal SpeD domain. Due to its relevance to the evolution of the class 2 eukaryotic AdoMetDC, a speD1speD2 fusion gene from the Lentisphaerota bacterium *Victivallis vadensis* [WP_116885763; 271 aa], which encodes a C-terminal SpeD homologous domain lacking at least two critical active site residues (Fig. S5), was also expressed in *E. coli* BL21*speD*. Expression of each fusion protein restored spermidine biosynthesis, confirming the presence of a *bona fide* AdoMetDC activity included in each (*D. phosphitoxidans* Figure 5, *Ca*. Omnitrophica bacterium Fig. S2, and *V. vadensis* Fig. S3).

**Figure 6:**
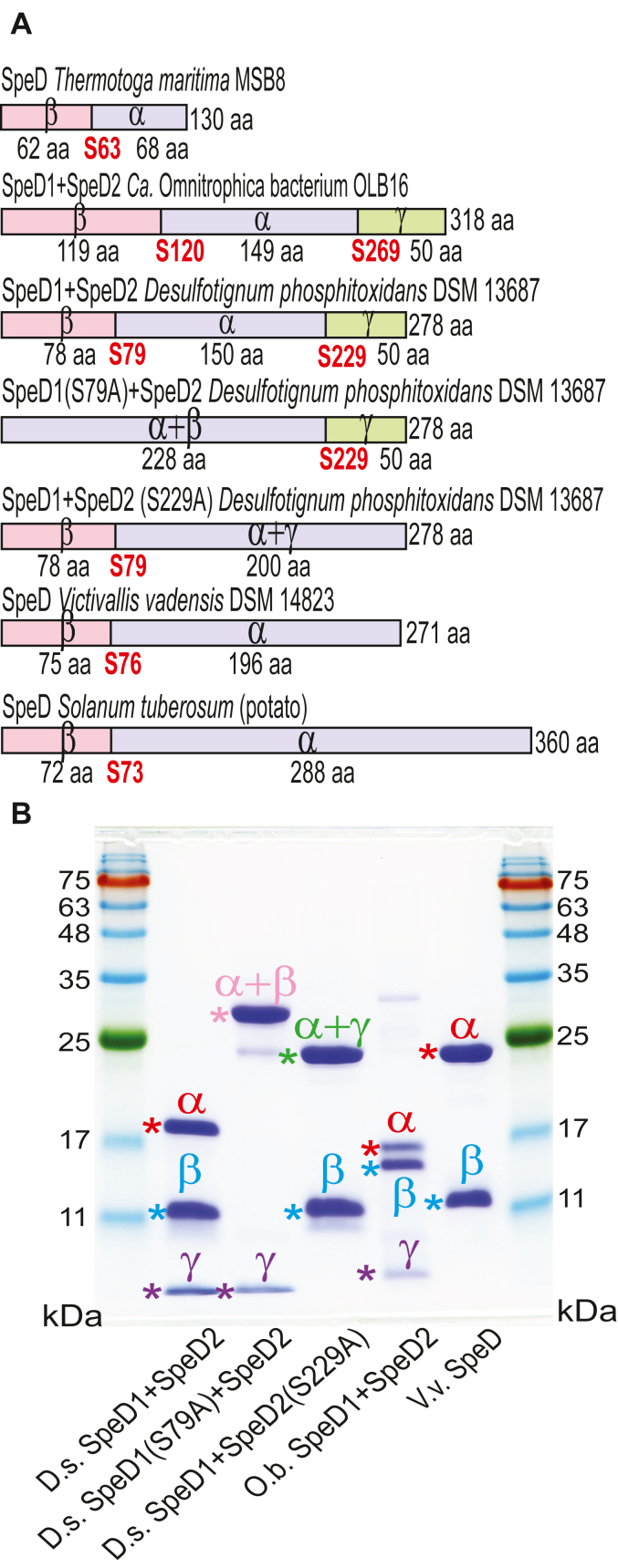
**SpeD-SpeDC fusion proteins.***A*, schematic representation of the proteins indicating the position of the serine residues predicted to be the autocatalytic self-cleavage sites that form the pyruvoyl cofactor. The different colors represent the predicted cleaved polypeptide subunits: α + β, α + β + γ, fused (α + β) + γ, β + fused (α + γ). *B*, purified proteins depicted in panel A after electrophoretic separation. D.s., *Desulfotignum phosphitoxidans* DSM 13687; O.b., Omnitrophica bacterium OLB16; V.v., *Victivallis vadensis* DSM 14823. Protein bands without an asterisk represent incompletely cleaved proenzyme protein or partially degraded protein. Colors indicate equivalent subunits.

The *Ca*. Omnitrophica bacterium and *D. phosphitoxidans speD1speD2* fusion ORFs were purified after expression in *E. coli* BL21 and their activity toward L-arginine, L-ornithine, and L-lysine determined (Table 4). No activity was detected with L-arginine or L-lysine but both proteins were active with L-ornithine, the *D. phosphitoxidans* and *Ca*. Omnitrophica SpeD1SpeD2 fusion proteins exhibiting a *k*_cat_/*K*_m_ for L-ornithine of 3600 M^−1^s^−1^, and 600 M^−1^s^−1^, respectively. The *D. phosphitoxidans* and *Ca*. Omnitrophica SpeD1SpeD2 fusion proteins are unprecedented in that they exhibit both AdoMetDC and ODC activities and are expected to contain two autocatalytic self-processing sites and produce two protein-derived pyruvoyl cofactors. Whereas the typical prokaryotic class 1b SpeD protein is processed into an α- and β-subunit, the *D. phosphitoxidans* and *Ca*. Omnitrophica SpeD1SpeD2 fusion proteins are predicted to be processed into α-, β-, and γ-subunits, with the pyruvoyl cofactors formed at the N-termini of the α- and γ-subunits (Fig. 6*A*).

**Table 4:** Kinetic constants determined for SpeD fusion proteins assayed with L-ornithine

Species	Protein	*K*_m_ (mM)	*k*_cat_ (s^−1^)	*k*_cat_/*K*_m_ (M^−1^s^−1^)
*Ca*. Omnitrophica bacterium SpeD1SpeD2	KXK35843	0.150 ± 0.014	0.10 ± 0.0020	600 ± 45
*Desulfotignum phosphitoxidans* SpeD1SpeD2	EMS77273	0.150 ± 0.010	0.53 ± 0.025	3600 ± 84
*D. phosphitoxidans* SpeD1SpeD2	EMS77273**(S79A)**	0.094 ± 0.0060	0.28 ± 0.011	3000 ± 30
*D. phosphitoxidans* SpeD1SpeD2	EMS77273 **(S229A)**	ND	ND	ND

No activity was detected with L-arginine or L-lysine as substrates. All assays performed in triplicate at 26 °C (mean ± S.D.).

Abbreviation: ND, no detectable activity.

To verify the position of the autocatalytic cleavage sites that form the pyruvoyl cofactor at the N-terminal serine of the processed subunits, we mutated to alanine the predicted processing site serines (S79A and S229A) in the *D. phosphitoxidans* SpeD1SpeD2 fusion protein (Fig. S5). The expected or known processing sites of the *D. phosphitoxidans* SpeD1SpeD2, SpeD1(S79A)SpeD2, and SpeD1SpeD2(S229A) fusion proteins, the *Ca*. Omnitrophica SpeD1SpeD2 fusion protein, the *V. vadensis* SpeD1/degradedSpeD2 fusion protein, the canonical class 1b SpeD protein of *Thermotoga maritima* (12) and the canonical eukaryotic class 2 AdoMetDC potato protein are depicted in Figure 6*A*. The fusion proteins were separated on an SDS-PAGE gel to visualize the processed subunits (Fig. 6*B*). Wildtype *D. phosphitoxidans* and *Ca*. Omnitrophica SpeD1SpeD2 fusion proteins are fully processed into the predicted α-, β- and γ-subunits, whereas the *V. vadensis* SpeD1/degradedSpeD2 fusion protein is processed into only two subunits.

For the *D. phosphitoxidans* SpeD1SpeD2 fusion protein, the N-terminal SpeD1 domain was confirmed as the *bona fide* AdoMetDC by expression of the S79A and S229A mutants in spermidine-deficient *E. coli* BL21*speD* (Fig. 5). Only the S229A mutant was able to restore spermidine biosynthesis, confirming the N-terminal SpeD1 domain as the functional AdoMetDC. The C-terminal SpeD2 domain was confirmed as the functional ODC by kinetic analysis of the catalytic properties of the purified S79A and S229A mutant proteins. Only the S79A protein exhibited L-ornithine decarboxylase activity with a *k*_cat_/*K*_m_ for L-ornithine of 3000 M^−1^s^−1^, similar to the native SpeD1SpeD2 protein (Table 4). We detected additional SpeD-SpeDC fusion proteins with a degraded C-terminal domain in the sulfate-reducing Desulfobacteria class of the Thermodesulfobacteriota phylum, for example, *Desulfobacter latus* (WP_178365596), which was previously assigned to the δ-Proteobacteria (39).

### Multiple independent emergences of ADC activity from SpeD homologs in bacteria and archaea

**Figure 7:**
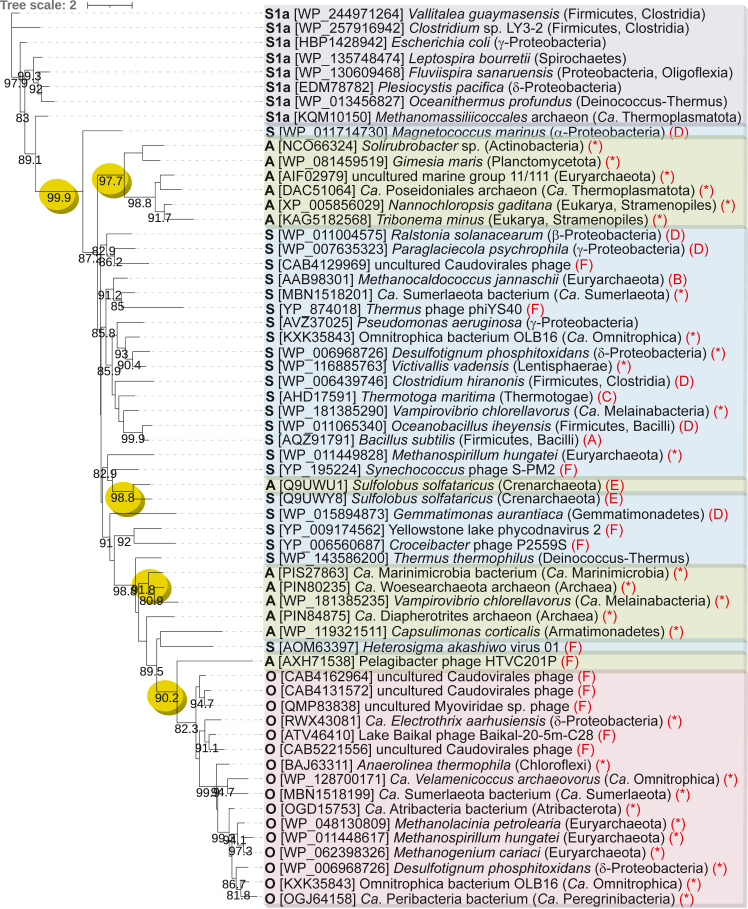
**Maximum likelihood phylogenetic tree of *S*-adenosylmethionine decarboxylase homologs.** Experimentally characterized enzymatic activity (*bold*, start of line): S, *bona fide S*-adenosylmethionine decarboxylase (*blue* background); A, L-arginine decarboxylase (*green* background), O, L-ornithine decarboxylase (*pink* background). Homologs of the *Escherichia coli* class 1a AdoMetDC (S1a) are shown in *mauve* background. Published enzymatic activities (end of line in *red*): A (59), B (60), C (12), D (46), E (21), F (20), ∗ current study. Bootstrap support above 80% is shown. Potential emergence points of new activities are shown by *yellow ellipses*. GenBank protein accession numbers are shown in *square brackets*; bacterial and archaeal phyla are shown in *parentheses*.

To determine whether ADC and ODC activities evolved on single or multiple occasions from AdoMetDC homologs in bacteria and archaea, a maximum likelihood phylogenetic tree was constructed of known *bona fide* SpeD proteins, and homologs with proven ODC or ADC activity (Fig. 7). Class 1a bacterial SpeDs were used as an out-group, although we note that they evolved from class 1b AdoMetDCs (40). The tree includes class 1b AdoMetDC homologs from bacteria, archaea, eukaryotes, viruses, and bacteriophages. It is notable that the bacterial, archaeal, and bacteriophage SpeDC ODCs are limited to a single well-supported clade containing only SpeDC proteins and the *Pelagibacter* phage HTVC201P SpeDA protein. They appear to have evolved only once from AdoMetDC followed by extensive *trans*-domain HGT between bacteria and archaea.

SpeDA ADCs have evolved from AdoMetDC at least three times. One highly supported clade of SpeDA proteins includes those from bacteria *Solirubrobacter* sp. (Actinomycetota phylum) and *G. maris* (Planctomycetota), uncultured marine archaea of marine group II/III and *Ca*. Poseidonales, and single-celled eukaryotes *N. nannochloropsis* and *T. minus* (both Stramenopiles phylum). This would suggest *trans*-domain horizontal gene transfer, between bacteria and archaea, and from bacteria or archaea to eukaryotes. A second emergence occurred in Crenarchaeota, *e.g.*, *S. solfataricus*, where the *bona fide* AdoMetDC exhibits high homology to the SpeDA ADC protein (21), suggesting a more recent gene duplication before neofunctionalization of one copy into ADC. A third emergence of SpeDA ADC proteins includes proteins from bacteria *Ca*. Marinibacteria bacterium (candidate phylum Marinimicrobia), *V. chlorellavorus* (Melainabacteria phylum), *C. corticalis* (Armatimonadota phylum), and from DPANN archaea *Ca*. Woesearchaeota and *Ca*. Diapherotrites. The phylogenetic tree suggests that the SpeDC proteins could have evolved from the Marinimicrobia clade of SpeDA proteins, rather than directly from AdoMetDC proteins. A potential fourth emergence includes the marine bacteriophage Pelagiphage HTVC201P, which is more similar to the SpeDC proteins.

**Figure 8:**
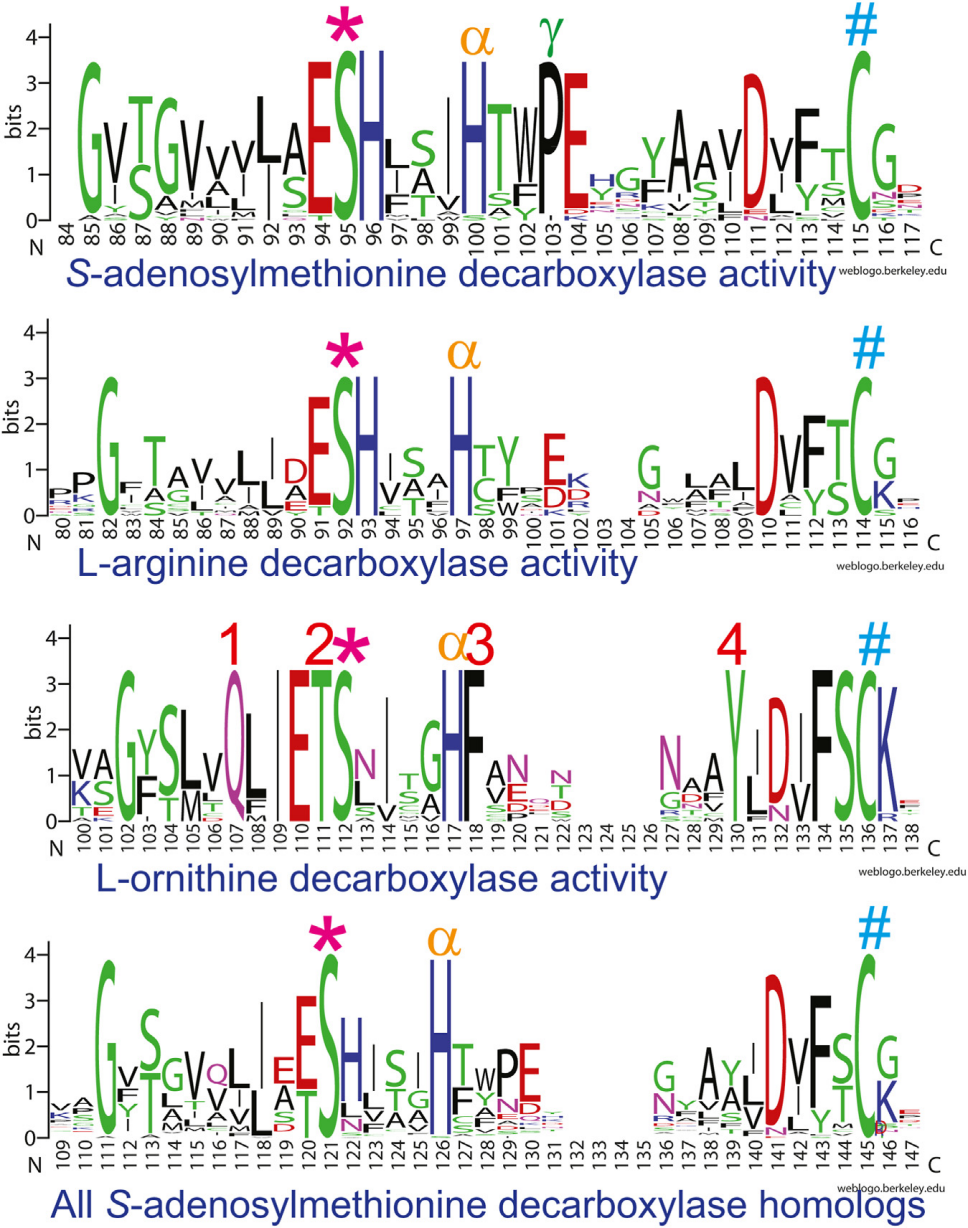
**WebLogo graphical representations of sequence alignments of the conserved central region of class 1b *S*-adenosylmethionine decarboxylase homologs.** Amino acids indicated by ∗, α and # are involved in autocatalytic cleavage and decarboxylation. L-ornithine decarboxylase (SpeDC) amino acids indicated by 1, 2, 3, and 4 are diagnostic for ODC identity. The proline indicated by γ distinguishes *bona fide* AdoMetDC from ADC.

The amino acids essential for autocatalytic self-processing of the *T. maritima* AdoMetDC proenzyme are S63, which forms the pyruvoyl cofactor, H68 and C83, which are also involved in catalysis (12). We reasoned that these amino acid positions would be conserved in SpeD, SpeDA, and SpeDC proteins but that substrate-binding residues would not. Proteins that possessed only *bona fide* AdoMetDC activity, only ODC activity, only ADC activity, and also all AdoMetDC homologs were aligned separately. The WebLogo representations of the central region of the proteins, where the processing and catalytic residues are found, are shown in Figure 8. This is the only region of the different forms of the AdoMetDC homologs that retains conserved residues. In the alignments, the equivalent of the *T. maritima* S63 is marked with ∗, H68 with α, and C83 with #. The biggest divergence from AdoMetDC is the SpeDC ODC group, where four conserved residues are diagnostic for this group: 1-Gln, 2-Thr, 3-Phe, and 4-Tyr (Fig. 8), and allow straightforward discrimination of this group from AdoMetDC and SpeDA ADC.

Using BLASTP with biochemically validated SpeDC proteins, the diagnostic amino acid positions indicate that there are at least 1,000 SpeDC homologs in bacteria and archaea. These include a large number of homologs from the bacterial Spirochaetota and Nitrospirota phyla, as well as the bacterial candidate phyla radiation, in addition to DPANN and Methanomicrobia archaea. In contrast, the SpeDA ADC group as a whole is relatively little diverged from the *bona fide* AdoMetDC, with the main difference being the lack of conservation of a Pro (marked γ) in the SpeDA proteins that is completely conserved in the *bona fide* AdoMetDCs. The lack of clear divergence of the SpeDA proteins from AdoMetDC may be due to the fact that there are at least four independent emergence events responsible for generating SpeDA proteins, which may confound phylogenetic signal that would allow discrimination between the two groups.

## Discussion

Diverse bacteriophages encode SpeD homologs that decarboxylate L-arginine (SpeDA) or L-ornithine (SpeDC) rather than AdoMet (SpeD) (20). It seemed unlikely that neofunctionalization of the SpeD homologs could have emerged while encoded by bacteriophages. Instead, it seemed more likely that the bacteriophage *speDA* and *speDC* genes, like bacteriophage *bona fide speD* genes were captured from ancestral bacterial hosts. Our systematic search for bacterial and archaeal neofunctionalized *speD* homologs that might be candidates for *speDA* or *speDC* genes uncovered an unsuspected metabolic subsystem of pyruvoyl-dependent ADC and ODC enzymes derived from AdoMetDC.

The pyruvoyl cofactor of AdoMetDC is subject to substrate-mediated transamidation resulting in the formation of alanine in place of pyruvate (11). Since this is dependent on the α-amine group of the substrate, it is also likely to occur with L-arginine and L-ornithine. Furthermore, purification of AdoMetDC in *E. coli* can lead to modification of the active site cysteine required for decarboxylation, which leads to irreversible inactivation (41). Taken together, these phenomena likely limit the observed efficiency of the pyruvoyl-dependent decarboxylation. Although the pyruvoyl-dependent ADCs and ODCs are considerably less efficient than their PLP-dependent nonhomologous isozymes, protein-derived pyruvoyl cofactors reduce dependency on expensive PLP and limited phosphate supply, and are also considerably smaller proteins. It is salient that in candidate phylum Omnitrophota, which includes *Ca. V. archaevorus* LiM and *Ca.* Omnitrophica bacterium OLB16, encoding SpeDC enzymes investigated in the current study, biosynthetic pathways for PLP are missing or incomplete across the phylum (42).

The original mechanism by which ADC and ODC enzymes emerged from AdoMetDC is likely to be gene duplication and subsequent neofunctionalization of one of the copies. However, HGT rather than vertical transmission appears to be the predominant mode of dissemination of the *speDA* and *speDC* genes among bacteria and archaea. It is notable that HGT may be particularly productive in spreading *speDA* and *speDC* genes among species of the bacterial candidate phyla radiation and DPANN archaea found in the same environment, for example, subsurface water.

Most of the *speDA* genes we investigated were found in gene clusters containing the *speY* gene for homospermidine biosynthesis, whereas the *speDC* genes were found in gene clusters with *speD*/*SpeE* or *casdh*/*casdc* for spermidine biosynthesis or *hss* for homospermidine biosynthesis. This suggests that the *speDC* gene may be able to integrate into diverse polyamine biosynthetic pathways more easily than *speDA* after HGT. This raises a number of questions such as how horizontally transferred *speDA* and *speDC* genes are integrated into existing polyamine biosynthetic pathways. Why are the pyruvoyl-dependent ADC and ODC enzymes able to replace PLP-dependent enzymes in receiving genomes? Is the reduction of dependency on PLP and phosphate a selective advantage?

The *speDA* gene encoding the production of agmatine from L-arginine would only be useful in a genome already encoding SpeB or AguA/AguB for conversion of agmatine to putrescine. That is, unless the *speDA* gene is horizontally transferred along with *speB* or *aguA*/*aguB*. The *speDC* gene, encoding direct production of putrescine from L-ornithine is not under this constraint. It is also possible that gene clusters encoding the entire polyamine biosynthetic pathway are horizontally transferred but such clusters would have to confer an adaptive advantage if the whole cluster replaces the preexisting host polyamine pathway. Besides reducing the requirement for PLP and phosphate, some of the gene clusters shown in Figure 2 would reduce dependency on AdoMet for polyamine biosynthesis. The production of homospermidine instead of spermidine requires only NAD+ as cofactor, and a SpeDC enzyme with SpeY or HSS requires only NAD+ instead of PLP and AdoMet that are required for the canonical *E. coli* spermidine biosynthetic pathway. It is unknown if homospermidine and spermidine play exactly the same role in bacterial physiology.

One of the intriguing features of the evolution of ADC and ODC from AdoMetDC is a retrograde evolution within an existing pathway. The emergence of ADC and ODC activities has not extended a preexisting pathway as is the case for the evolution of spermine synthase from spermidine synthase (17, 18). It has not modified a metabolic intermediate toward a new function, such as the evolution of putrescine *N*-methyltransferase from spermidine synthase (43). Instead, the emergence of ADC and ODC activities from AdoMetDC has sent the neofunctionalized enzyme backward to an earlier step in the biosynthetic pathway. The phylogenetically diverse SpeDA and SpeDC enzymes represent another example of the plasticity of bacterial and archaeal polyamine metabolism (16). It is likely that other examples of unsuspected substrate-shifted homologs exist in prokaryotic polyamine metabolism. Although metabolic plasticity is indicated by the shift from recognition of the aminocarboxypropyl group of dcAdoMet to the aminocarboxybutyl groups of L-arginine and L-ornithine (Fig. 4), the aminocarboxylpentyl group of L-lysine is not recognized by any of the AdoMetDC homologs, in contrast to PLP-dependent L-ornithine decarboxylases of the alanine racemase and aspartate aminotransferase folds, which can exhibit a relatively efficient recognition of the L-lysine aminocarboxypentyl group (44, 45).

The SpeD-SpeDC fusion proteins encoded by the δ-proteobacterium *D. phosphitoxidans* and the *Ca*. Omnitrophica bacterium OLB16 are unprecedented in possessing two autocatalytic self-cleaving sites in the same polypeptide chain that generate two internal serine-derived pyruvoyl cofactors. Unless the SpeD domain is differentially controlled by putrescine levels produced by the SpeDC domain, then putrescine and spermidine biosynthesis would be coregulated. The homologous fusion protein encoded by Lentisphaerota bacterium *V. vadensis* has lost critical active site residues in the C-terminal SpeDC domain and consequently cannot self-cleave and so lacks ODC activity.

The existence of the ODC activity-lacking *V. vadensis* SpeD-SpeDC fusion protein suggests an evolutionary scenario for the emergence of the eukaryotic class 2 AdoMetDC. Although the eukaryotic class 2 AdoMetDC exhibits almost no detectable sequence homology to the prokaryotic class 1b SpeD protein, structural analysis previously revealed that the eukaryotic AdoMetDC structure is composed of two class 1b polypeptides fused together, with the C-terminal domain lacking a self-cleavage site, and consequently lacking a C-terminal pyruvoyl cofactor (12). There are some bacteria that encode a class 2 AdoMetDC but phylogenetic analysis indicates that these genes were originally acquired from a eukaryotic source (46). The class 2 AdoMetDC appears to be a eukaryotic invention that was present in the last eukaryotic ancestor. It is thought that the class 2 AdoMetDC was originally composed of two fused *bona fide* SpeD proteins; however, such a fusion does not seem to be extant in prokaryotes. It is more plausible that the original fusion protein was a SpeD-SpeDC fusion protein with defunct SpeDC domain like the *V. vadensis* fusion protein.

**Figure 9:**
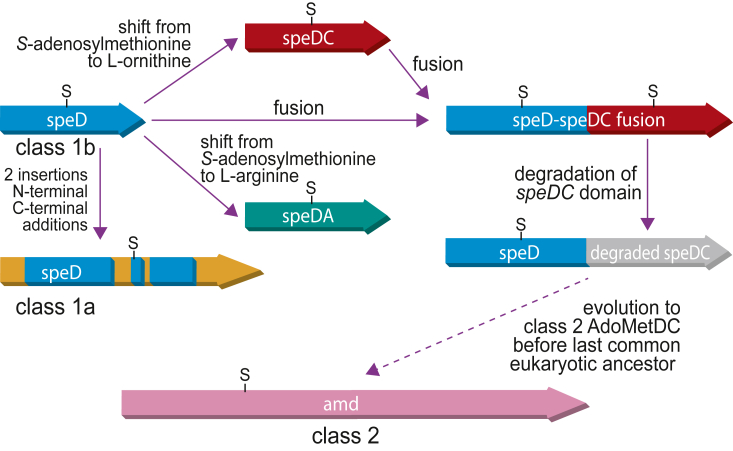
**A hypothetical model for the evolution of eukaryotic class 2 AdoMetDC from bacterial class 1b AdoMetDC.** Not shown, it is formally possible that speDC arose from speDA. Genes: *speD*, *S*-adenosylmethionine decarboxylase; *speDA*, L-arginine decarboxylase; speDC, L-ornithine decarboxylase; *amd*, eukaryotic class 2 *S*-adenosylmethionine decarboxylase.

We present a hypothetical model for the evolution of the different AdoMetDC structural and activity variants in Figure 9. The original AdoMetDC was probably the prokaryotic class 1b form. Amino acid insertions into and extensions of the class 1b form resulted in the prokaryotic class 1a form. Gene duplication of the class 1b *speD* and neofunctionalization resulted in *speDA* and *speDC*. It is formally possible that *speDC* arose from *speDA* rather than directly from *speD*. Fusion of *speD* and *speDC* genes resulted in a bifunctional enzyme; subsequently SpeDC pyruvoyl formation and ODC activity was lost in some of the Lentisphaerota-related lineage. The fusion protein of two SpeD homologous domains would have been present in either the archaeal host or α-proteobacterial ancestor of the mitochondrion that formed the first eukaryotic cell. However, we did not detect a SpeD fusion protein encoded in extant archaea or α-proteobacteria.

A generally accepted explanation for the origin of eukaryotes is the merger of an archaeal host, likely an Asgardarchaeota cell with an α-proteobacterium (47). The Lentisphaerota and *Ca*. Omnitrophica phyla are related to the Planctomycetota phylum as part of the extend polyvinyl chloride superphylum (48). Our model for the evolution of eukaryotic class 2 AdoMeDC, based on highly similar fusion proteins encoded by numerous Lentisphaerota and *Ca*. Omnitrophica genomes, is more easily explained with the available evidence by the single domain (bacterial) origin of eukaryotes model based on a Planctomycetota-like ancestor (49, 50). We also identified SpeD-SpeDC fusion proteins with a degraded C-terminal SpeDC domain in the Desulfobacterales, currently classified as being in the Thermodesulfobacteriota phylum but previously assigned to the δ-proteobacterial class (39). This would fit with the alternative scenario for evolution of eukaryotes (the Syntrophy hypothesis) where a sulfate-reducing δ-proteobacterium took up an endosymbiotic Asgard archaeal cell that became the nucleus and that symbiotic complex then took up an α-proteobacterial cell which became the mitochondrion (51). With the available prokaryotic genome sequences, our hypothesis for the evolution of the eukaryotic class 2 AdoMetDC does not currently fit with the simple Asgard/α-proteobacteria model. During the period from the first to the last eukaryotic common ancestor, the sequence of the AdoMetDC fusion protein would have radically changed to the extent that little amino acid sequence similarity remained with the original prokaryotic version, in direct contrast to the highly conserved AdoMetDC obligatory partner enzyme spermidine synthase (7).

## Experimental procedures

### Bacterial strains and genes

The *E. coli* strain BL21 (DE3) (Invitrogen) was used for expression of proteins that were then purified. Construction of the BL21Δ*speD* strain was described previously (52). All genes were synthesized by GenScript and cloned into pET28b-TEV and pETDuet-1 in the Nde1 and Xho1 sites except for Marinimicrobia bacterium CG08_8_20_14_0_20_45_22 gene encoding PIS27863, which was cloned into pET28b-TEV in the BamH1 and HindIII sites.

### Protein purification

Target genes were expressed from pET28a-TEV in *E. coli* BL21 (DE3) as described previously (20). A single colony of BL21 (DE3) containing the target gene was inoculated into 40 ml of LB medium with 50 μg/ml kanamycin and grown overnight at 37 °C. Twenty-five milliliters of the overnight culture was then inoculated into 2 l of LB medium with 50 μg/ml kanamycin, and grown to an *A*_600_ of approximately 0.4. At this point, 0.2 mM IPTG was added to induce expression overnight at 16 °C, and the culture was then centrifuged to harvest cells. The cell pellet was resuspended in buffer A (20 mM Tris–HCl buffer (pH 7.5), 500 mM NaCl, 10 mM imidazole, 0.02% Brij 35, and 1 mM tris(2-carboxyethyl)phosphine (disulfide reducing agent)). A cell disruptor was used to lyse the cells at 10,000 p.s.i., and this was repeated three times. Cell lysate was obtained by centrifuging the debris, and the lysate was then applied to a His-tag Ni column (Hi-trap HP column from Cytiva Life Science) at 1 ml/min, then washed with 1% buffer B (20 mM Tris–HCl (pH 7.5), 500 mM NaCl, 800 mM imidazole, 0.02% Brij 35 and 1 mM tris(2-carboxyethyl)phosphine), for a total volume of 50 ml. The His-tagged protein was eluted using 20 column volumes of a gradient 0 to 50% buffer B at a rate of 3 ml/min. The elution tubes were assessed by separation on an SDS-PAGE gel, and the tubes containing the tagged protein were pooled and concentrated using an Amicon Ultra-15 centrifugal filter to a volume of less than 3 ml. This was then dialyzed against 20 mM Tris–HCl buffer (pH 7.5), 500 mM NaCl, 0.02% Brij 35, and 5% glycerol at 4 °C overnight.

### Protein concentration determination

The AdoMetDC/SpeD protein is produced as a proenzyme that is then autocatalytically cleaved into subunits. Not all AdoMetDC proteins are completely cleaved into the corresponding subunits. Total protein, which might include some unprocessed proenzyme and processed subunits, was determined using a BioTek plate reader Gen5 with the program Protein 280, and protein samples and control standard were loaded in quadruplicate into a Take3 micro-volume plate. Absorbance was measured at 280, 260 and 320 nm, and the protein extinction coefficient and molecular weight of the protein was used to determine protein concentration. Proteins that were not fully cleaved (as determined by SDS-PAGE gel analysis) were imaged on the gel, and ImageJ (https://imagej.nih.gov/ij/) was used to calculate the percentage of processed protein relative to the unprocessed proenzyme band, and the percentage of processed protein was taken as the final concentration of active enzyme.

### Decarboxylase activity analysis

Enzyme activity assays (200 μl) were performed in Corning Costar flat bottom 96-well plates in a buffer consisting of 50 mM Hepes pH 7.7, 100 mM NaCl, and 1 mM dithiothreitol to which was added 150 μl of carbon dioxide assay stable test solution from Diazyme labs, Inc. Between 0 to 10 mM L-arginine, L-ornithine, or L-lysine was added, and tested with different concentrations of enzymes. After mixing, the 96-well plate was placed in the BioTek plate reader reading a wavelength of *A*_340_, 10 s/read for 40 min, with set temperature at 26 °C. Data from the plate reader which fitted a linear model was used to calculate reaction rate. The reaction rate mean velocity V (Absorbance Units (AU)/min) is derived from the linear slope automatically by the plate reader software, and an extinction coefficient of 6.349 AU/mM/cm was used to calculate mM NADH/min (53). Using the GraphPad Prism Michaelis–Menten model graph fitting, the *K*_m_ and V_max_ were obtained, and then the enzyme concentration was included to calculate *k*_cat_. The concentration of each protein used in the assays is shown in Table S1. Substrate saturation curves used for the assays from selected enzymes are shown in Fig. S6.

### Polyamine analysis by LC-MS

Polyamines were extracted from *E. coli* cells as described previously (20). Briefly, harvested cells were resuspended in 200 μl of polyamine extraction buffer (100 mM Mops pH 8.0, 50 mM NaCl, and 20 mM MgCl_2_), frozen in liquid N_2_ and thawed in a 37 °C water bath and repeated three times. Sixty microliters of 40% trichloroacetic acid was added, place on ice for 10 min, and centrifuged to separate the supernatant containing the polyamines. To the supernatant was added 1 ml of 2 M NaOH and 10 μl benzoyl chloride followed by mixing for 2 min on a vortex mixer, and then placement in a chemical hood at room temperature for 60 min. Two milliliters of saturated NaCl was then added, mixed for 2 min, and then 2 ml of diethyl ether added, mixed again for 2 min, and left in a chemical hood for 30 min. The upper layer of diethyl ether containing the polyamines was placed into LC-MS analysis vials and left in a chemical hood until all the diethyl ether had evaporated. Fully dried samples were then analyzed by LC-MS on an Agilent 1290 Infinity HPLC system using an Eclipse XDB-C18 column (4.6 × 150 mm, 5 μm particle size) that was coupled to an Agilent 6130 quadrapole electrospray ionization mass spectrometer run in positive mode with a scan range of 100 to 1100 m/z. Liquid chromatography was carried out at a flow rate of 0.5 ml/min at 20 °C with a 5 μl injection volume using a gradient elution. The solvent systems used were solvent A, HPLC grade water, 0.1% v/v formic acid and solvent B, HPLC grade acetonitrile 0.1% v/v formic acid. The gradient started with 30% solvent A/70% solvent B, rising to 10% solvent A/90% solvent B in 9 min, then maintaining for 3 min.

### Genomic and phylogenomic analyses

Homologs of class 1b AdoMetDC/SpeD were identified in bacterial genomes using BLASTP, PSIBLAST, and TBLASTN at the National Center for Biotechnology Information with diverse bacterial, archaeal, and bacteriophage class 1b SpeD query proteins. Protein alignments were performed using MUSCLE (https://www.ebi.ac.uk/Tools/msa/muscle/) (54). Maximum Likelihood phylogenetic trees based on the amino acid alignments created by MUSCLE were constructed using IQ-TREE (http://iqtree.cibiv.univie.ac.at/) (55) employing default parameters for protein alignments and the automatic substitution model with 1000 ultrafast bootstrap analysis (56). Maximum Likelihood trees were visualized using iTOL (https://itol.embl.de/upload.cgi) (57). Sequence logos were created with WebLogo (https://weblogo.berkeley.edu/) (58).

## Data availability

All data are contained within the article and the Supplementary material.

## Supporting information

This article contains supporting information.

## Conflict of interest

The authors declare that they have no conflicts of interest with the contents of this article.
