# Supplementary material for: Neofunctionalization of S-adenosylmethionine decarboxylase into pyruvoyl-dependent L-ornithine and L-arginine decarboxylases is widespread in bacteria and archaea
Source: J Biol Chem. 2023 Jul 1;299(8):105005. doi: 10.1016/j.jbc.2023.105005 (PMC10407285; doi:10.1016/j.jbc.2023.105005)
Supplement: Supporting Figures S1–S6 and Table S1 [file mmc1.pdf]

Supplementary Information

Neofunctionalization of *S*-adenosylmethionine decarboxylase into pyruvoyl-dependent L-ornithine and L-arginine decarboxylases is widespread in bacteria and archaea

Bin Li[1], Jue Liang[1], Margaret A. Phillips[1], Anthony J. Michael[1*]

[1]Department of Biochemistry, UT Southwestern Medical Center, Dallas, Texas, USA

Corresponding author: Anthony J. Michael

[*]E-mail: anthony.michael@utsouthwestern.edu

Table S1. Protein concentrations of *S*-adenosylmethionine decarboxylase (AdoMetDC/SpeD) homologs in enzyme assays

AdoMetDC (SpeD) homologs assayed with L-arginine

Species	protein	Enzyme conc. (µM)
Ca. Diapherotrites archaeon	PIN84875	0.17
Ca. Woesearchaeota archaeon	PIN80235	0.36
Vampirovibrio chlorellavorus	WP_181385235	0.18
Capsulimonas corticalis	WP_119321511	0.50
Ca. Poseidonales archaeon	DAC51064	0.20
Unc. marine group II/III euryarchaeote	AIF02979	0.68
Gimesia maris	WP_081459519	0.32
Solirubrobacter sp.	NCO66324	1.59
Tribonema minus	KAG5182568	0.64
Nannochloropsis gaditana	XP_005856029	0.80
Ca. Marinimicrobia bacterium	PIS27863	0.32

AdoMetDC (SpeD) homologs assayed with L-ornithine

Species	protein	Enzyme conc. (µM)
Ca. Kaiserbacteria bacterium	OGG79367	1.10
Methanogenium cariaci JCM 10550	WP_062398326	1.14
Ca. Sumerlaeota bacterium	MBN1868927	2.46
Ca. Electrothrix aarhusiensis	RWX43081	1.79
Methanolacinia petrolearia DSM 11571	WP_048130809	0.96
Methanospirillum hungatei JF-1	WP_011448617	1.57
Ca. Velamenicoccus archaeovorus LiM	WP_128700171	2.97
Ca. Peribacteria bacterium	OGJ64158	0.55
Ca. Atribacteria bacterium	PIU25646	0.48
Anaerolinea thermophila UNI-1	BAJ63311	0.62

AdoMetDC (SpeD) homolog fusion proteins assayed with L-ornithine

Species	protein	Enzyme conc. (µM)
Ca. Omnitrophica bacterium SpeD1SpeD2	KXK35843	0.66
Desulfotignum phosphitoxidans SpeD1SpeD2	EMS77273	0.20
D. phosphitoxidans SpeD1SpeD2	EMS77273**(S79A)**	0.29

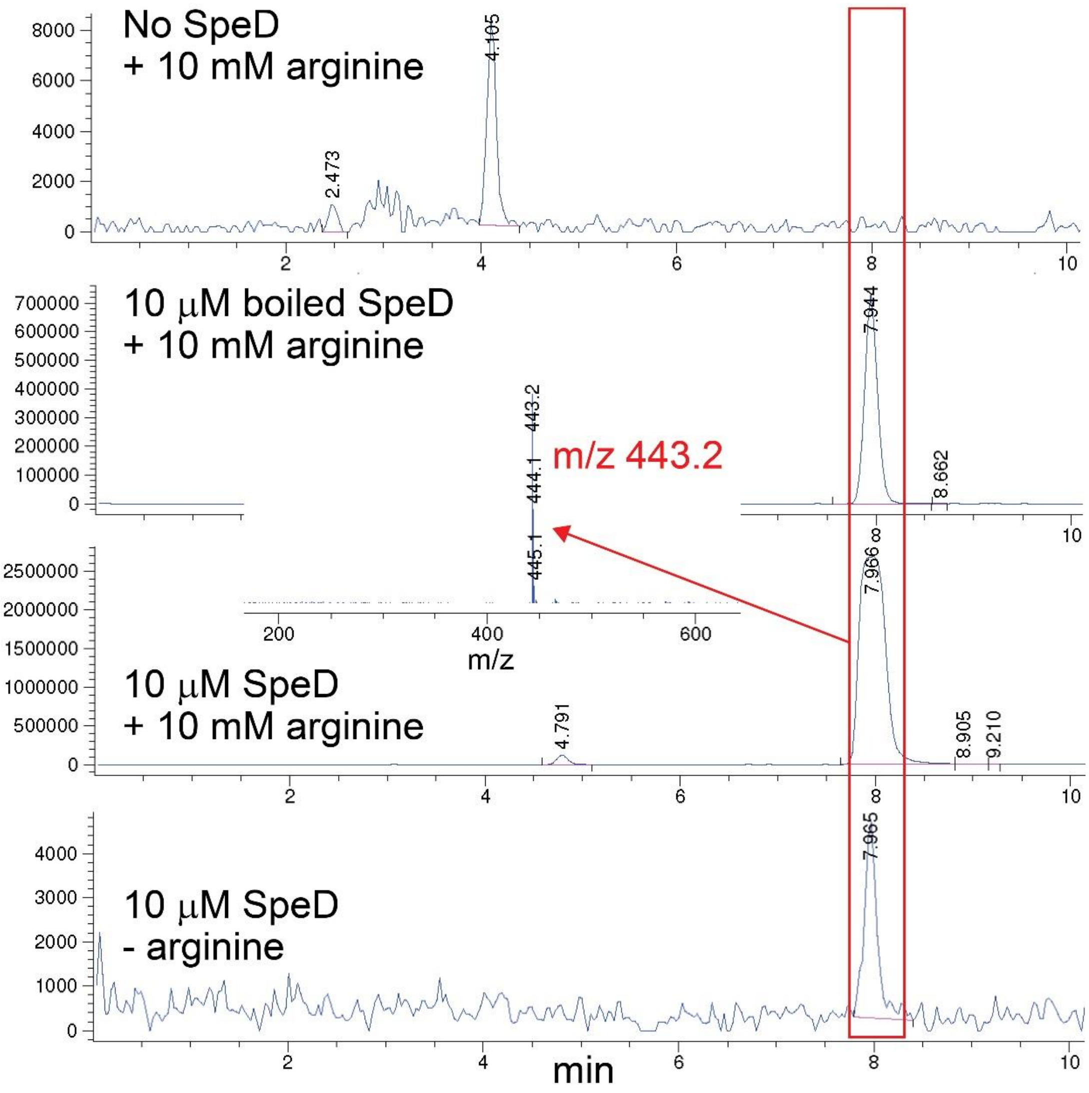

Figure S1. LC-MS analysis of products of *in vitro* assay of Marinimicrobia bacterium AdoMetDC/SpeD homologue with L-arginine. Assay was performed with 100 mM HEPES pH 7.8 and 1 mM DTT at 27°C for 30 min, and reaction products were then benzoylated. Shown are Extracted Ion Chromatograms (EICs) for tribenzoylated agmatine (EIC, 442.9:443.9) with the tribenzoylated agmatine peak highlighted in red. Insert shows mass spectrum for peak at approximately 7.96 min indicating a prominent mass for tribenzoylated agmatine (m/z = 443.2).

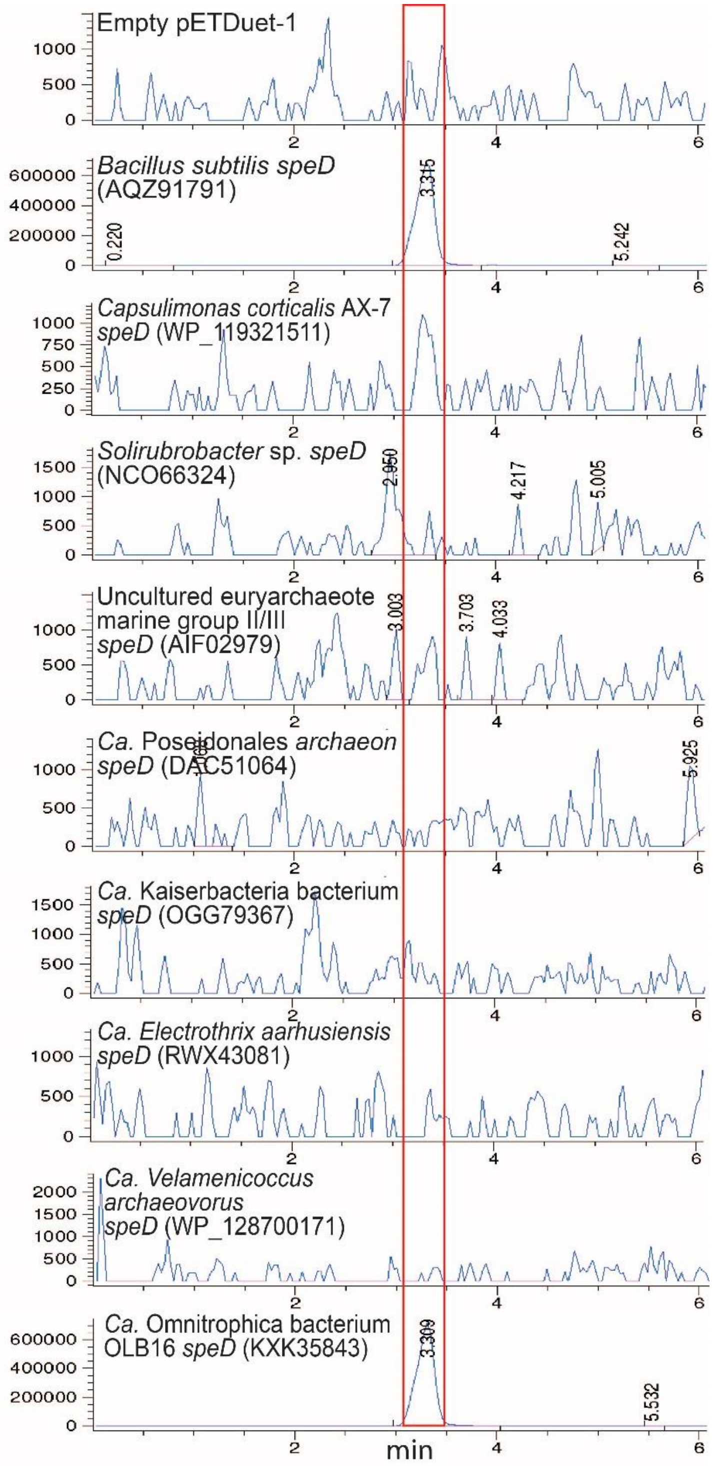

Figure S2. LC-MS analysis of benzoylated cell extracts of *E. coli* BL21Δ*speD* expressing *speD* homologues. Benzoylated extracts of *E. coli* BL21Δ*speD*, an *E. coli* strain lacking *S*-adenosylmethionine decarboxylase (*speD*), expressing either an empty pETDuet-1 plasmid or diverse *speD* homologs. Encoded SpeD homologous protein Genbank accession numbers are indicated in parentheses. The tribenzoylated spermidine peak is highlighted in red.

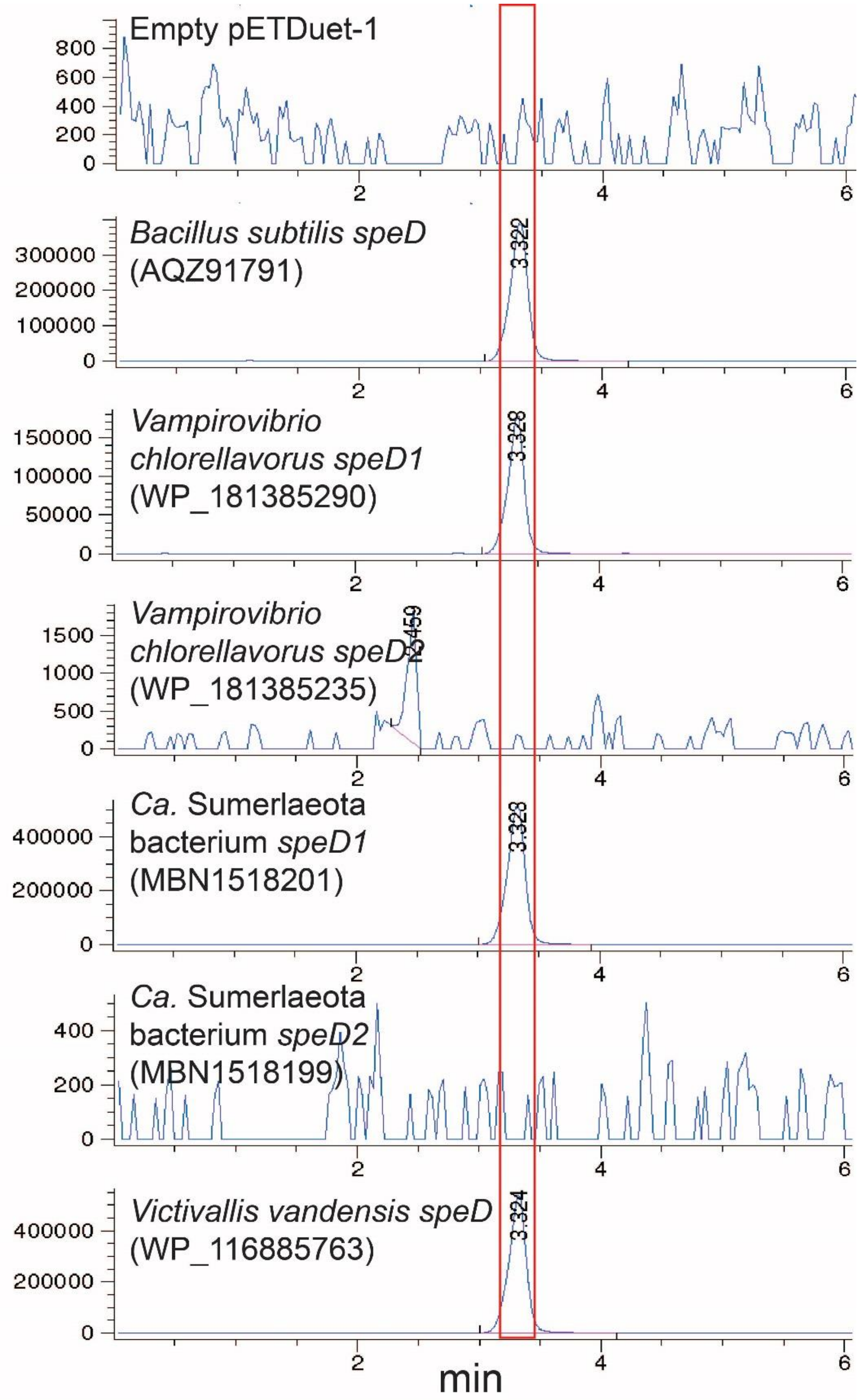

Figure S3. LC-MS analysis of benzoylated cell extracts of *E. coli* BL21Δ*speD* expressing *speD* homologues. Benzoylated extracts of *E. coli* BL21Δ*speD*, an *E. coli* strain lacking *S*-adenosylmethionine decarboxylase (*speD*), expressing either an empty pETDuet-1 plasmid or diverse *speD* homologs. Encoded SpeD homologous protein Genbank accession numbers are indicated in parentheses. The tribenzoylated spermidine peak is highlighted in red.

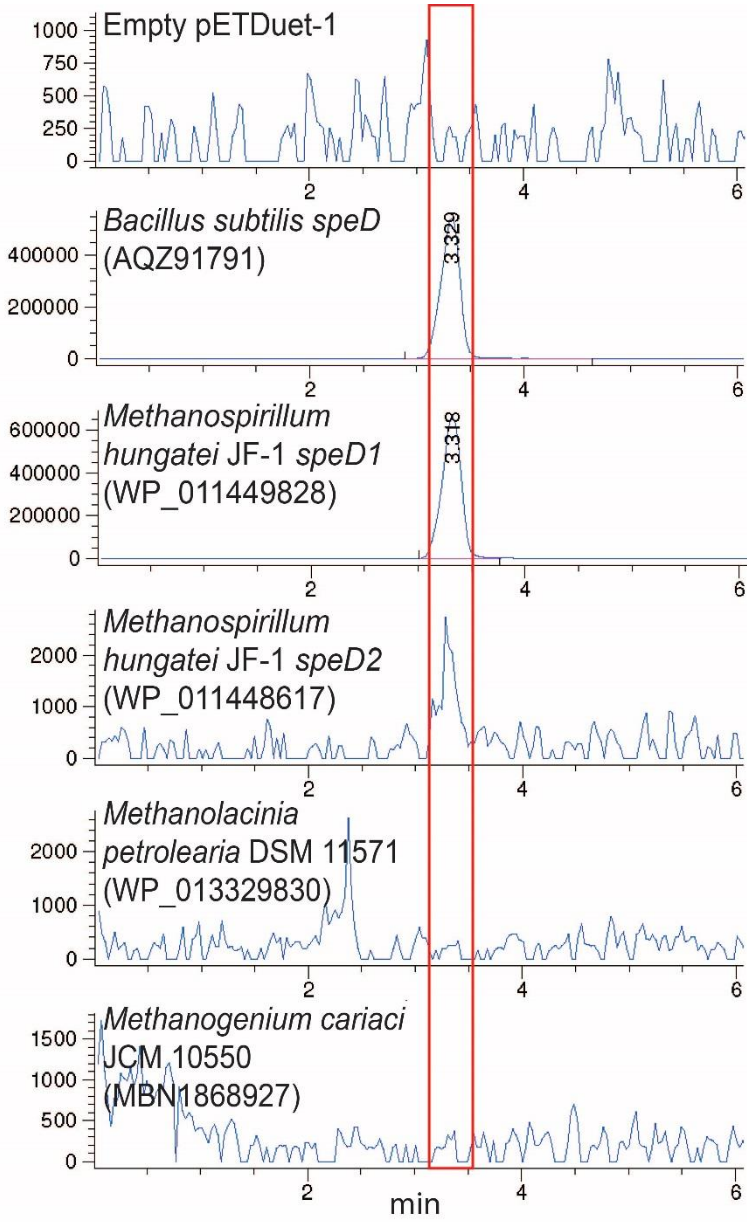

Figure S4. LC-MS analysis of benzoylated cell extracts of *E. coli* BL21Δ*speD* expressing *speD* homologues. Benzoylated extracts of *E. coli* BL21Δ*speD*, an *E. coli* strain lacking *S*-adenosylmethionine decarboxylase (*speD*), expressing either an empty pETDuet-1 plasmid or diverse *speD* homologs. Encoded SpeD homologous protein Genbank accession numbers are indicated in parentheses. The tribenzoylated spermidine peak is highlighted in red.

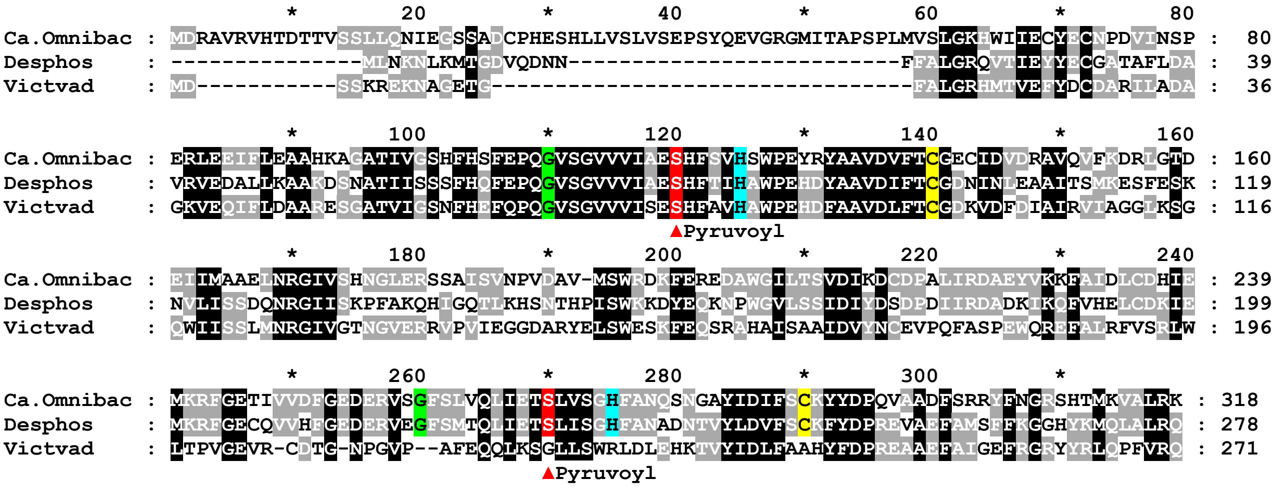

Figure S5. Alignment of SpeD1SpeD2 fusion proteins. *Ca.*Omnibac, *Ca*. Omnitrophica bacterium OLB16 [KXK35843]; Desphos, *Desulfotignum phosphitoxidans* [WP_006968726]; Victvad, *Victivallis vadensis* [WP_116885763]. The glycine residues in green, serine in red, histidine in blue and cysteine in yellow are conserved in all functional SpeD homologues. Autocatalytic self-cleavage occurs at the serine residue in red, which becomes the pyruvoyl cofactor after processing.

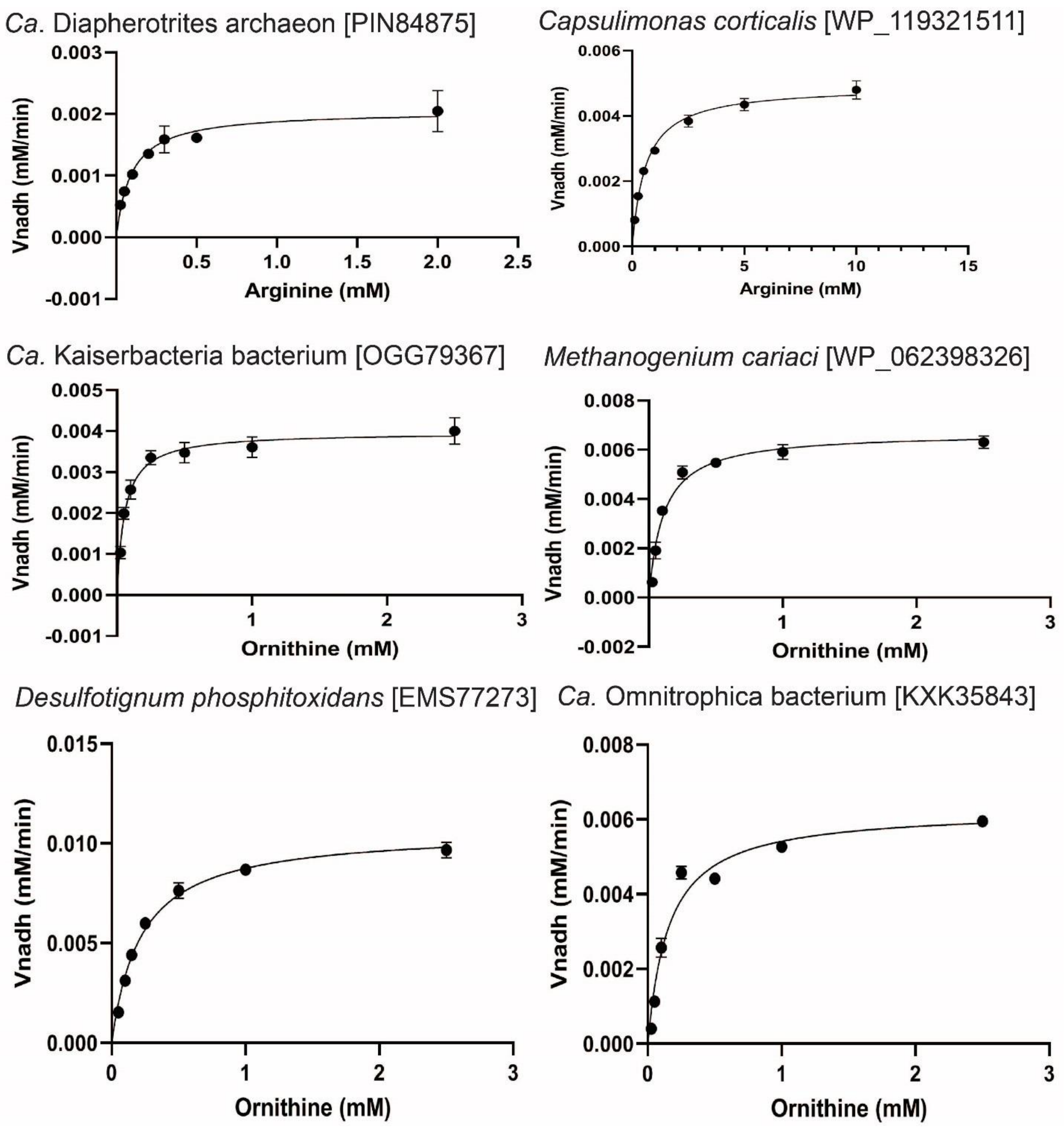

Figure S6. Substrate saturation curves for selected AdoMetDC/SpeD homologs. The assay conditions are described in the Experimental procedures. The y-axis represents the rate of NADH oxidation to NAD+ measured with the CO_2 detection kit.
